# Transverse testicular ectopia associated with incarcerated inguinal hernia: a case report

**DOI:** 10.1186/1757-1626-1-200

**Published:** 2008-09-30

**Authors:** Duygu Tatli, Kemal Varim Numanoglu

**Affiliations:** 1 MD, Assist. Dr. Department of Pediatric Surgery, School of Medicine, Zonguldak Karaelmas University, Kozlu, Zonguldak, Turkey; 2 MD, Assist. Prof. Department of Pediatric Surgery, School of Medicine, Zonguldak Karaelmas University, Kozlu, Zonguldak, Turkey

## Abstract

Transverse testicular ectopia is rarely associated with incarcerated inguinal hernia. A 14-month-old male complaint of irreducible inguinal hernia due to transverse testicular ectopia is reported. The clinical and operative findings and treatment options are discussed. It is thought that surgeons who frequently repair inguinal hernias should be aware of the appropriate surgical management options available to them when this condition is unexpectedly identified during inguinal exploration.

## Background

Transverse testicular ectopia (TTE) is a rare scrotal anomaly occurring consequent to the migration of both gonads to the same hemiscrotum. The ectopic testis may be located on the inner inguinal ring, in the inguinal canal, or in the contralateral hemiscrotum.[1,2] Although seen characteristically as an ipsilateral inguinal hernia and contralateral undescended testis, the diagnosis can be established only during surgical exploration.

Hereby, a case has been presented, which had been pre-diagnosed as having an incarcerated inguinal hernia, and taken into operation in which transverse testicular ectopia was detected.

## Case report

A 14-month-old male was admitted with the complaints of vomiting and painful right inguinal swelling. This swelling had been present for about one month, but in the last 8 hours, the swelling had become pronounced and tender.

**Figure 1:**
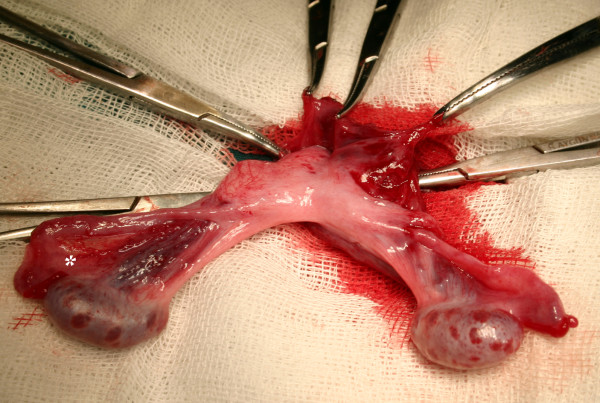
**Operative wiew during right inguinal exploration.** All structures delivered through right groin incision as seen. Asterisk (*) denotes incarcerated gonad.

On physical examination, there was an incarcerated inguinal hernia in the right groin. The left testis could not be palpated in the scrotum or the inguinal canal. The patient was taken to the operation room as soon as appropriate preoperative preparations had been completed. At the time of operation, right inguinal region explored. Hernia sac and cord structures traversing the internal inguinal ring were identified. Unexpectedly, a second testis with a well-grown vas deferens and testicular vessels was found incarcerated at the level of the internal ring (Fig. 1). There were two vas deferenses and vascular structures accompanying each gonad. Following medial elongation of the incision, the existence of Müllerian remnants was eliminated. Following hernia repair, the ectopic gonad was fixed to the opposite hemiscrotum through a transseptal route.

No complications occurred in the postoperative period. Abdominal ultrasound and the voiding cystourethrogram revealed normal findings.

## Discussion

Although rare, transverse testicular ectopia, occurring secondary to the migration of both testes to the same hemiscrotum, is a well-known congenital anomaly. This finding was first reported by Lenhossek in 1886, as an autopsy finding.[3] In the following years, over 100 cases have been reported.

The embryologic etiology of TTE is controversial. Several theories such as adhesion and fusion of developing Wolffian canals, aberrant gubernaculum, testicular adhesions, defective formation of the internal inguinal ring and traction on a testis by persistent müllerian structures have been suggested. Persistent müllerian duct structures (PMDS) may result from the failure of synthesis or releases of müllerian duct inhibitory factor (MIF), the failure of end organs to respond to MIF, or defect in the timing of the release of MIF. It seems possible that the mechanical effect of the persistent müllerian duct structures prevents the testicular descent or leads to both testicles descending toward the same hemiscrotum, producing TTE.[4] The association with cryptorchidism is accompanied by an increase in malignancy potential of crossed ectopic testes with malignancy rates similar to undescended testes.

Transverse testicular ectopias are classified into three types according to the existence of various additional anomalies:[1]

1. Observed only with inguinal hernia (40–50%).

2. Observed with persistent or rudimentary Mullerian duct structures (30%).

3. Observed with additional anomalies other than Mullerian remnants (inguinal hernia, hypospadias, pseudohermaphroditism, and scrotal anomalies) (20%).

TTE patients are generally admitted due to inguinal hernia on the migrated side of the ectopic testis or an undescended testis on the opposite side. However, there have only been four cases admitted with incarceration as in the present case. [5-8]

In many cases, the diagnosis may be established during the operation. However, there are also articles reporting that preoperative diagnosis may be established by ultrasound, computerized tomography, magnetic resonance imaging or magnetic resonance venography.[9]

The treatment of transverse testicular ectopia is focused on the detection of associated congenital abnormalities and placement of ectopic testicles into anatomical positions. This preserves fertility and allows monitoring for the development of malignancy. If two gonads come into view during exploration of one inguinal side, complete abdominal exploration must be performed. Intraoperative intraabdominal evaluation via mini laparotomy or transinguinal diagnostic laparoscopy allows for detection of müllerian structures and genitourinary congenital abnormalities. A biopsy should be taken from tissue remnants between cord structures or abdomen. There is no report of malignancy arising from the retained müllerian structures, and the absence of MIF does not appear to increase the relative risk of testicular malignancy. Therefore, routine hysterectomy is not recommended in patients who have obvious uterus and fallopian tubes. Extensive dissection of vas deferenses and excision of persistent müllerian duct structures should be avoided to prevent the injury.[4]

Transseptal orchiopexy or extra-peritoneal transposition of the testis is the treatment of choice. Long-term follow-up should be performed as the possibility of infertility and malignant transformation is rather high.[10]

Transverse testicular ectopia, characteristically accompanied by ipsilateral inguinal hernia, and a contralateral undescended testis, is a rare anomaly. The present case is the fifth case in the literature admitted with findings of incarceration and described after emergent surgery. Surgeons who frequently repair inguinal hernias should be aware of the appropriate surgical management options available to them when this condition is unexpectedly identified during inguinal exploration. It is thought that it will be beneficial to keep this rare clinical entity in mind, in cases of incarcerated inguinal hernia with contralateral undescended testis.

## Consent

Written informed consent was obtained from the patient's parents for publication of this case report and accompanying images. A copy of the written consent is available for review by the Editor-in-Chief of this journal.

## Competing interests

The authors declare that they have no competing interests.

## Authors' contributions

DT and KVN were involved in the treatment of the patient and wrote and finalized the manuscript. All authors read and approved the final manuscript.
